# Recent developments in strawberry aroma: Essential aroma-active components and their formation mechanisms, influencing factors, and analytical technologies

**DOI:** 10.1016/j.fochx.2026.104710

**Published:** 2026-10-29

**Authors:** Su Xu, Jinjin Jiang, Guangcan Tao, Qiang Fei, Lingshuai Meng, Qinglong Meng, Liang Zhao

**Affiliations:** a School of Food Science and Engineering, Guizhou Engineering Research Center for Characteristic Flavor Perception and Quality Control of Drug-Food Homologous Resources, Guiyang University, Guiyang 550005, China; b National Engineering Research Center for Fruit and Vegetable Processing, Key Laboratory of Fruit and Vegetable Processing, Key Laboratory of Food Non-Thermal Processing, Ministry of Agricultural and Rural Affairs, College of Food Science & Nutritional Engineering, China Agricultural University, Beijing 100083, China

**Keywords:** Strawberries, Aromas, Metabolic pathways, Regulatory mechanisms, Influencing factors

## Abstract

Strawberries are valued for their sweet-sour taste, color, and aroma. Dynamic changes in aromatic compounds affect aroma characteristics across ripening, storage, and processing, often leading to aroma loss or deterioration. Revealing aroma formation mechanisms and regulatory strategies is crucial. This review integrates aroma identification techniques with multi-omics to systematically analyze aroma-active compounds in strawberries, elucidating their formation and transformation. It summarizes key influencing factors and advanced analytical methods, constructing a framework that links the chemical basis to regulatory pathways. Future research should prioritize the functional characterization of key genes and the identification of regulatory targets for expression, as well as the development of environmentally friendly and efficient preservation and processing technologies. These efforts aim to maximize the retention of natural aroma components, providing a scientific foundation for maintaining the characteristic aroma of strawberries and enhancing their overall quality.

## Highlights

• Esters, lactones, aldehydes, furanones, terpenes, sulfur compounds are main aromas.
• Fatty acid, amino acid, carbohydrate, terpenoid metabolism pathways create aromas.
• Affecting factors including gene, cultivation, postharvest, maturity are reviewed.
• Analysis technologies and multi-omics approaches for aroma detection are reviewed.

## 1 Introduction

Strawberry (*Fragaria × ananassa*) is a perennial evergreen herbaceous plant belonging to the *Rosaceae* family. Its fruit is characterized by a fragrant aroma, juicy texture, and a balanced sweet-sour flavor, contributing to its high nutritional value. Often referred to as the “Queen of Fruits,” strawberries are highly favored by consumers worldwide. They not only hold a significant position in the fresh food market but also serve as a key raw material in the food processing industry (Xu, Shi, et al., 2024). The scale of the strawberry industry continues to expand both domestically and internationally, making the cultivation of high-quality seeds and the enhancement of their quality a central focus for industry development. The flavor quality of strawberries, particularly the aroma components, directly influences their market appeal and economic value. Consequently, researching the mechanisms and regulations governing the formation of strawberry aroma is of considerable scientific significance and holds promising application prospects (Zhang et al., 2025).

Strawberry aroma is a crucial component of fruit flavor quality, primarily composed of volatile substances such as esters (Cozzolino et al., 2022; Fang et al., 2024; Rey-Serra et al., 2022), terpenes (Xiaofen et al., 2011), aldehydes (Liu et al., 2024), furans (Gu et al., 2022), and sulfur-containing compounds (Du, Song, & Rouseff, 2011). Despite their small proportion in the fruit, the types, proportions, and dynamic changes of these substances directly influence the sensory quality and consumer preferences of strawberries (Schwieterman et al., 2014; Ulrich & Olbricht, 2016). From an industrial perspective, strawberries are susceptible to softening and spoilage post-harvest, leading to a rapid loss of aroma and negatively impacting market circulation and the quality of processed products. Consequently, investigating the formation mechanisms, influencing factors, and regulation techniques of strawberry aroma is significant for extending shelf life, enhancing processing adaptability, and developing high-quality varieties.

As shown in Fig. 1, recent researches on strawberry aroma have primarily concentrated on the following aspects: (1) composition and identification of aroma compounds. More than 360 volatile compounds have been identified in strawberries, with esters being the predominant components contributing to the fruity and sweet characteristics, such as ethyl acetate, ethyl butyrate, and ethyl hexanoate (Urun et al., 2021). Additionally, compounds like *γ*-dodecanone and *γ*-decalactone impart a peach-like aroma (Cao et al., 2024). Aldehyde compounds, including hexanal, heptanal, and octanal, significantly contribute to the fruity and grassy aromas of strawberries (Prat et al., 2014). Additionally, terpenes such as linalool, d-limonene, and naringenin impart a complex floral and fruity aroma to strawberries (Du, Plotto, et al., 2011). Furanone compounds, such as 4-methoxy-2,5-dimethyl-3(2H)-furanone, impart creamy and fruity aromas to strawberries (Fan et al., 2021). (2) The factors influencing aroma formation include varietal differences, genotypes, environmental factors, cultivation conditions, fruit maturity, post-harvest storage conditions and processing methods. (3) The synthesis pathways and regulatory mechanisms involved in aroma compound formation primarily occur through the fatty acid pathway, amino acid pathway, terpene pathway, and carbohydrate pathway. Additionally, research has identified that phenylpropanoid metabolism and alpha-linolenic acid metabolism pathways play crucial roles in the aroma development of cream-flavored strawberry varieties (Fang et al., 2024). (4) Innovations in analytical techniques, such as headspace solid-phase microextraction coupled with gas chromatography–mass spectrometry (HS-SPME-GC–MS), as well as transcriptome and metabolome integration analyses, have been extensively utilized for the identification and dynamic monitoring of strawberry aroma compounds (Wang et al., 2022). With advancements in omics technology and molecular biology, research on strawberry aroma is transitioning from component identification to exploration of the underlying formation mechanisms. Concurrently, the extraction and analytical techniques for bonded aroma compounds have been continuously refined, establishing a robust foundation for the classification of aroma types across different strawberry varieties. Nevertheless, many unknown molecular mechanisms governing the synthesis and metabolism of aroma compounds remain, and a systematic analysis of the regulatory network is still required.

**Fig. 1:**
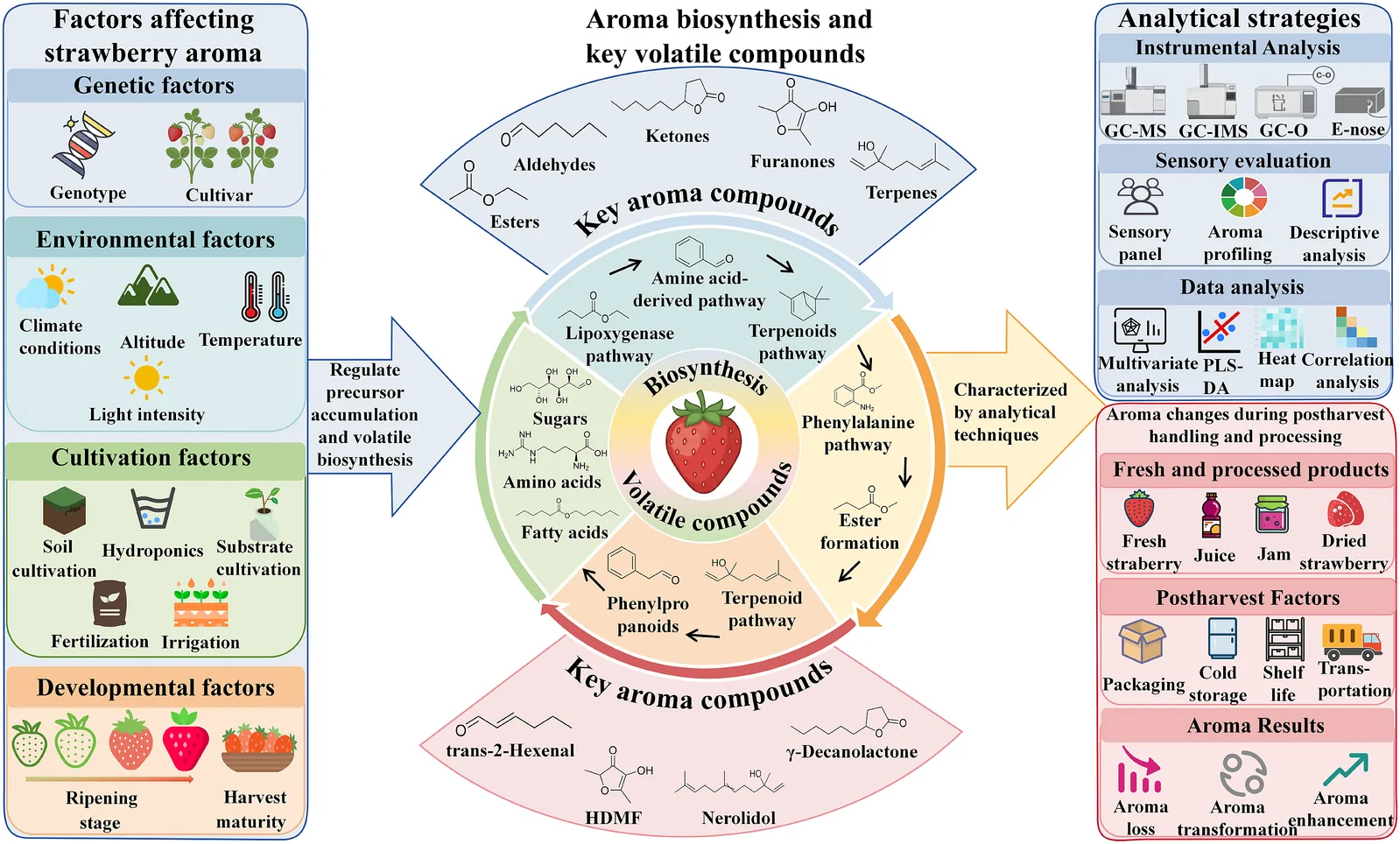
Strawberry aroma: affect factors, biosynthesis pathways, analytical strategies, and changes during postharvest handling and processing. HDMF: 4-Hydroxy-2,5-dimethyl-3(2H)-furanone; GC–MS: Gas Chromatography-Mass Spectrometry; GC-IMS: Gas Chromatography-Ion Mobility Spectrometry; GC-O: Gas Chromatography-Olfactometry; E-nose:Electronic Nose; PLS-DA: Partial Least Squares Discriminant Analysis.

Future research should focus more on the key genes involved in aroma formation, the regulatory nodes of metabolic pathways, and the influence of external factors and gene interactions on aroma. By integrating multiple omics approaches and developing novel storage methods and processing technologies, new strategies can be devised for strawberry flavor breeding and quality control, ultimately fostering the high-quality development of the strawberry industry.

## 2 The main volatile aroma components in strawberries

The aroma of strawberries consists of a complex mixture of volatile organic compounds (VOCs), which include esters, lactones, aldehydes, furanones, terpenes, and sulfur-containing compounds. However, the detection or relative abundance of a volatile compound does not necessarily indicate its contribution to the overall aroma perception (Abouelenein et al., 2023; Rey-Serra et al., 2022). The sensory relevance of individual volatiles largely depends on their concentrations in relation to their odor detection thresholds (OTs), defined as the minimum concentrations at which their odors can be perceived under specified conditions. The odor activity value (OAV), calculated as the ratio of a compound's concentration to its corresponding OT (OAV = concentration/OT), is widely employed to estimate its potential sensory contribution (Kim et al., 2013; Yan et al., 2018). Compounds with OAVs greater than 1 are generally considered potentially aroma-active, while those with lower values may still influence the overall aroma through interactions with other odorants (Rey-Serra et al., 2022; Xiaofen et al., 2011). Nevertheless, OAVs should be interpreted with caution, as odor thresholds can vary depending on the food matrix and experimental conditions, and OAVs alone cannot fully account for perceptual interactions within complex mixtures (Schwieterman et al., 2014; Sheng et al., 2021; Xiaofen et al., 2011). Consequently, evidence from gas chromatography-olfactometry (GC-O), aroma recombination, and omission experiments can provide complementary support for identifying key odorants (Xu et al., 2026). In light of these considerations, the following sections focus on representative volatile compounds with documented sensory relevance, rather than offering an exhaustive description of all detected volatiles. Their characteristic odor attributes and available evidence of aroma activity are summarized in Table 1.

**Table 1:** Main volatile aroma compounds in strawberries.

Type	Compounds	Molecular formula	Structural formula	Odor description	CAS	OT (mg/kg)	References
Esters	Ethyl acetate	C_4_H_8_O_2_		Fruity, brandy, pineapple-like	141–78-6	0.005	(Liu et al., 2024; Xiaofen et al., 2011)
	Hexyl acetate	C_8_H_16_O_2_		Apple, cherry, pear, floral, sweetish	142–92-7	0.115	(Liu et al., 2024; Sheng et al., 2021)
	Octyl acetate	C_10_H_20_O_2_		Neroli, jasmine, fruity	112–14-1	0.047	(Dong et al., 2013)
	Methyl butyrate	C_5_H_10_O_2_		Fruity, apple, banana, pineapple, ester-like, green	623–42-7	0.059	(Sheng et al., 2021; Song et al., 2017; Xiaofen et al., 2011)
	Ethyl butyrate	C_6_H_12_O_2_		Fruity, sweet, pineapple	105–54-4	0.0009	(Sheng et al., 2021; Song et al., 2017)
	Hexyl butyrate	C_10_H_20_O_2_		Fruity, apricot	2639-63-6	0.203	(Dong et al., 2013)
	Octyl butyrate	C_12_H_24_O_2_		Herbaceous, green, orange, parsley, melon	110–39-4	0.25	(Dong et al., 2013)
	Methyl hexanoate	C_7_H_14_O_2_		Fruity, ether-like, pineapple，herbaceous	106–70-7	0.07	(Dong et al., 2013; Sheng et al., 2021)
	Ethyl hexanoate	C_8_H_16_O_2_		Fruity, pineapple, banana, green apple, winy	123–66-0	0.005	(Dong et al., 2013; Sheng et al., 2021)
	Hexyl hexanoate	C_12_H_24_O_2_		Herbaceous	6378-65-0	6.4	(Dong et al., 2013)
	Methyl o-aminobenzoate	C_8_H_9_NO_2_		floral,sweet, grape-like	134–20-3	0.003	(Pillet et al., 2017; Song et al., 2017)
Lactones	γ -decanolactone	C_10_H_18_O_2_		Peach, cream, coconut	706–14-9	0.0011	(Cao et al., 2024; Fan et al., 2021; Oh et al., 2021)
	δ -cinelactone	C_8_H_14_O_2_		Creamy, coconut, oily texture	698–76-0	0.2	(Cao et al., 2024)
	δ-decanolactone	C_10_H_18_O_2_		Creamy, peaches, nut-like	705–86-2	0.066	(Cao et al., 2024)
Aldehydes	Hexanal	C_6_H_12_O		Grassy, green vegetables, astringent	66–25-1	0.005	(Liu et al., 2024;Prat et al., 2014; Schwieterman et al., 2014; Sheng et al., 2021)
	trans-2-Hexenal	C_6_H_10_O		Green note, herbaceous	6728-26-3	0.0885	(Prat et al., 2014; Schwieterman et al., 2014; Sheng et al., 2021)
	Octanal	C_8_H_16_O		Citrus, fatty,floral	124–13-0	0.000587	(Song et al., 2017)
	Nonanal	C_9_H_18_O		Citrusy, rose-like,waxy	124–19-6	0.0011	(Song et al., 2017)
	Benzaldehyde	C_7_H_6_O		bitter almond, cherry-like, nutty	100–52-7	0.75089	(Song et al., 2017)
	Phenylacetaldehyde	C_8_H_8_O		Sweet, honey-like, rose-like	122–78-1	0.0063	(Song et al., 2017)
Furanones	HDMF 4-Hydroxy-2,5-dimethyl-3(2H)-furanone	C_6_H_8_O_3_		Sweet,caramel-like,pineapple-like	3658-77-3	0.0223	(Schwieterman et al., 2014; Song et al., 2016)
	DMMF 2,5-Dimethyl-4-methoxy-3(2H)-furanone	C_7_H_10_O_3_		Sweet,floral,mango-like,pineapple-like, ether-like	4077–47–8	3.4	(Schwieterman et al., 2014; Zhang et al., 2025)
Terpenes	Linalool	C_10_H_18_O		Floral,sweet,citrus-like,ether-like	78–70-6	0.00022	(Barbey et al., 2021; Schwieterman et al., 2014; Sheng et al., 2021; Urrutia et al., 2017)
	Nerolidol	C_15_H_26_O		woody-floral,weet	7212-44-4	2.25–10	(Barbey et al., 2021; Schwieterman et al., 2014)
	Geraniol	C_10_H_18_O		Rosaceous floral, sweet, citrus, honey-like	106–24-1	0.0011	(Liu et al., 2024; Ulrich & Olbricht, 2016)
	α-Pinene	C_10_H_16_		Woody,resinous-like, pine needle-like	80–56-8	0.014	(Ulrich & Olbricht, 2016)
	Caryophyllene	C_15_H_24_		Woody,peppery, pungent, herbaceous	87–44-5	0.064	(Barbey et al., 2021; Madera et al., 2026)
	α-Farnesene	C_15_H_24_		Waxy,floral, herbaceous	502–61-4	NA	(Fan et al., 2022; Madera et al., 2026)
Sulfur compounds	Methanethiol	CH_4_S		Sweet, vegetal, onion-like, garlic-like	74–93-1	0.0002	(Du, Song, & Rouseff, 2011; Yan et al., 2018)
	Dimethyl Sulfide	C_2_H_6_S		Sweet,corn-like	75–18-3	0.00012	(Du et al., 2012)
	Methyl thioacetate	C_3_H_6_OS		Sweet, sulfurous, mango-like, pineapple-like	1534-08-3	0.23(AIR)	(Du et al., 2012; Du, Song, & Rouseff, 2011)
	Methyl Thiobutyrate	C_5_H_10_OS		Fruity,pineapple-like	2432-51-1	0.14(AIR)	(Du et al., 2012; Du, Song, & Rouseff, 2011)

Note: The odor descriptions are derived from the FEMA database; OT (odor threshold) represents the odor threshold of the compound in water (mg/kg), the data is from “Compilation of Odor Thresholds of Compounds” (Leo Van Hemert, Beijing, Science Press, 2015). Odor threshold values cited in this review were primarily determined in water matrix, and values may vary depending on the matrix and methodology.

### 2.1 Esters and lactones

The key volatile components detected in strawberries are primarily esters. Esters constitute approximately 40% to 62.5% of the total volatile substances in strawberries, serving as the predominant contributors to the aroma composition. Due to their relatively low odor thresholds, these compounds play a crucial role in shaping the overall flavor profile of strawberries (Liu et al., 2024). From a sensory perspective, esters are closely associated with floral and fruity aroma (Song et al., 2017). Previous studies indicate that the volatile esters present in fresh strawberries are predominantly short- and medium-chain esters, which significantly contribute to the development of aromas such as orange blossom, gardenia, apple, and cherry (Dong et al., 2013). The main contributing esters include hexyl acetate, characterized by aromas of apple, cherry, pear, floral, and sweet; methyl butyrate, which exhibits aromas of apple, banana, pineapple, ester-like, and green; ethyl butyrate, primarily noted for its fruity, sweet, and pineapple aromas; methyl caproate, which has fruity, ether-like, and pineapple-like characteristics; and ethyl caproate, which presents a complex aroma of wine, pineapple, banana, fruit flavor, and green apple (Bian et al., 2023; Dong et al., 2013; Sheng et al., 2021; Song et al., 2017). Collectively, these esters form the foundational framework of strawberry aroma. In addition to the common esters, methyl ortho-aminobenzoate (MA) is a distinctive aroma component found in wild strawberries, imparting a robust spicy aroma. This compound is a key aroma component that differentiates wild strawberries from their cultivated counterparts (Pillet et al., 2017). In strawberry varieties exhibiting a broader spectrum of aroma types, lactone compounds significantly contribute to the development of aroma profiles. These compounds typically emit scents reminiscent of coconut and milk (Song et al., 2023). Notably, gamma-decalactone is characterized by its pronounced peach, cream, and coconut fragrances, while delta-decalactone is associated with cream and coconut aromas. Additionally, delta-octalactone presents a complex aroma that combines cream, peach, and nut notes (Song et al., 2023). In *Fragaria nilgerrensis Schlecht.*, a wild diploid strawberry species, this group of lactones is present in high concentrations and plays a crucial role in establishing its distinctive peach-like aroma (Wang et al., 2022).

### 2.2 Aldehydes

Aldehydes represent a crucial class of volatile aromatic compounds in strawberries, with their biosynthesis primarily occurring through two metabolic pathways: the lipoxygenase (LOX) pathway and the amino acid conversion pathway. In the LOX pathway, aldehydes utilize unsaturated fatty acids, such as linolenic and linoleic acids, as substrates to generate hydroperoxides, catalyzed by lipoxygenase. These hydroperoxides are subsequently oxidized and cleaved into short-chain C6 or C9 aldehydes by hydroperoxide lyase (HPL) (Ullah et al., 2024). Research indicates that the expression of key genes, including *FaLOX5* and *FaLOX9*, is positively correlated with C6 aldehyde content (Lubes & Goodarzi, 2017; Stolterfoht et al., 2019). C6 aldehydes are the predominant aromatic components in immature strawberries, with 2-hexenal and (*E*)-3-hexenal contributing significantly to the green or fresh aroma of strawberries (Du, Plotto, et al., 2011). In the “Hongyan” variety, the increase in the level of (*E*)-2-hexenal gives rise to the “green grass” and “green leaves” scents, which are the main sources of the green aroma during the early stage of its maturity (Zhang et al., 2025). An additional pathway for aldehyde synthesis is the amino acid conversion pathway. This pathway involves the conversion of branched-chain amino acids, catalyzed by branched-chain amino acid transaminases and branched-chain *α*-keto acid dehydrogenase complexes, to produce aldehyde precursor *α*-keto acids. These *α*-keto acids then undergo decarboxylation and oxidation reactions to yield aldehyde compounds. For instance, phenylalanine is transformed into cinnamic acid by phenylalanine ammonia lyase (PAL) and is further metabolized into benzaldehyde or vanillin (Gonda et al., 2018). Benzaldehyde imparts aromas of “almond” and “cherry” and is a significant aromatic compound in strawberries, while vanillin bestows the “Oriental Beauty” variety with aromas of “vanilla” and “cream,” becoming the core component of its creamy scent (Fang et al., 2024).

### 2.3 Furanones

Furanones are key aroma compounds in strawberry fruit. Among these, 4-Hydroxy-2,5-dimethyl-3(2H)-furanone (HDMF), 2,5-Dimethyl-4-hydroxy-3(2H)-furanone (DMHF), and 2,5-Dimethyl-4-methoxy-3(2H)-furanone (DMMF) have been identified as the principal volatile organic compounds contributing to strawberry aroma (Negri et al., 2015; Song et al., 2016). These compounds exhibit diverse aroma characteristics at varying concentrations: under low concentration conditions, HDMF is typically described as “fruity,” while DMHF is characterized as either “fruity” or “sweet.” In contrast, at high concentrations, HDMF and DMMF display “caramel flavor” and “rose aroma,” respectively (Lu et al., 2021). From a biosynthetic perspective, the synthesis of HDMF initiates with the fructose metabolite fructose-1,6-diphosphate, which is enzymatically converted into the intermediate acetolactate, ultimately cyclizing to form HDMF. The key catalytic enzyme in this pathway is quinone oxidoreductase (FaQR), which directly regulates HDMF synthesis and serves as the rate-limiting enzyme for furanone biosynthesis in strawberries. Furthermore, HMMF, a direct precursor of HDMF, is converted into HDMF with aromatic activity under the catalysis of FaQR (Li, Zhang, et al., 2021; Yamada et al., 2019). Subsequently, HDMF is further catalyzed by *O*-methyltransferase (FaOMT) and methoxylated to yield DMMF (Badovskaya et al., 2021). In strawberry varieties such as “Rose Princess,” DMMF serves as the primary aroma component, imparting the fruit with both “caramel aroma” and “floral aroma” (Badovskaya et al., 2021).

### 2.4 Terpenes

Terpene compounds are essential components of the volatile organic compounds found in strawberries, primarily encompassing monoterpenes (C10), sesquiterpenes (C15), and triterpenes (C30) (Madera et al., 2026). The monoterpenes identified in strawberries include linalool, geraniol, *α*-terpinene, and *α*-pinene, while the sesquiterpenes comprise nerolidol, *β*-isoamylene, and caryophyllene (Madera et al., 2026; Urrutia et al., 2017). Notably, linalool and nerolidol exhibit high odor activity values (OAV > 1) and are recognized as representative terpene components. *Cinnamomum camphora* emits aromas characterized as “fragrant,” “floral,” and “citrus,” and is widely distributed across varieties such as “General” and “Hongyan.” In contrast, nerolidol presents aromas reminiscent of “rose” and “apple,” predominantly found in varieties such as “Snow White” (Chen et al., 2023). The study mapped the synthesis of terpenoids such as linalool and nerolidol to a major quantitative trait locus (QTL) region in 3B linkage group and confirmed the presence of a terpenoid synthesis gene cluster within this locus, which participates in regulating aroma formation through synergistic expression. The expression of gene *FanNES1* is not only controlled by its own expression QTL, but also significantly positively correlated with the accumulation levels of various terpenoids (Barbey et al., 2021). Further research has shown that the nerolidol synthase FaNES1 in cultivated strawberries is a key enzyme in the synthesis of linalool and nerolidol. The functional expression of this enzyme can significantly increase the content of linalool in cultivated and wild varieties, and linalool and *α*-pinene begin to accumulate in the immature stage of the fruit. In addition, the synthesis process is directly regulated by transcription factor complexes *FaMYB98* and *FaERF9*, which activate the terpene synthesis pathway by binding to the terpene synthase (TPS) gene promoter region (Al-Salihi & Ford, 2023). In wild strawberries, relevant studies have also identified monoterpene synthase, which can catalyze the generation of monoterpenes such as *α*-pinene, *β*-carvacene, and *α*-terpenene (Asaph et al., 2004). It is worth noting that in addition to imparting aroma, some terpene compounds also exhibit biological activity. In addition to being a key aroma component, linalool has also been reported to have antibacterial properties and may regulate gut microbiota composition and related inflammatory factors through ingestion (Tan et al., 2024).

### 2.5 Sulfur-containing compounds

Although sulfur-containing volatile compounds are generally less abundant than esters and other major volatile classes, their low odor thresholds render them significant contributors to the aroma of strawberries. These compounds primarily consist of thiols, sulfides, and thioesters, including methanethiol, dimethyl sulfide, dimethyl disulfide and dimethyl trisulfide (Dordevic et al., 2023; Porter et al., 2023). Their sensory contributions vary depending on the identity and concentration of the compounds. Methanethiol has been identified as a potent odorant in ripe strawberries and is associated with green sensory attributes, while *S*-methyl thiobutanoate (butanethioic acid, *S*-methyl ester) has been linked to overripe and fermented notes (Fan et al., 2021; Porter et al., 2023). The sensory potency of these compounds is further underscored by the reported aqueous odor detection thresholds of approximately 30 ng/L for dimethyl disulfide and 10 ng/L for dimethyl trisulfide (Pochiraju et al., 2021). However, it is important to note that these values are reported for water rather than strawberry fruit, and their direct application to the assessment of strawberry aroma necessitates caution, as odor perception is influenced by the surrounding matrix.

The composition of sulfur-containing volatiles changes during processing, potentially altering the balance between desirable fruity notes and undesirable sulfurous odors. Tekgül and Erten (2022) reported that dimethyl sulfide, which was not detected in fresh samples, became one of the predominant headspace volatiles in hot-air-dried strawberries, contributing to cabbage-like and sulfurous odor characteristics (Tekgül & Erten, 2022). More recently, Yao et al. (2025) investigated aroma deterioration during the thermal sterilization of strawberry juice and identified dimethyl trisulfide at 3.61 μg/kg as a major contributor to cooked-cabbage off-flavor, supported by aroma recombination and omission experiments (Yao et al., 2025). These findings indicate that thermal processing can promote the formation or accumulation of certain sulfur volatiles while simultaneously reducing the abundance of compounds responsible for fresh and fruity aromas. Consequently, the changes in sulfur-containing compounds depend on the processing conditions and the chemical characteristics of individual odorants rather than following a uniform pattern of degradation. Further investigations, combining targeted quantification with sensory validation, are needed to clarify the stability and transformation of individual sulfur volatiles, particularly thioesters, during strawberry processing.

## 3 The formation mechanisms of strawberry aromas

Fig. 2 illustrates the essential metabolic pathways involved in the synthesis of key aroma volatiles in strawberries, including fatty acid metabolism, amino acid metabolism, terpenoid metabolism, and carbohydrate metabolism.

**Fig. 2:**
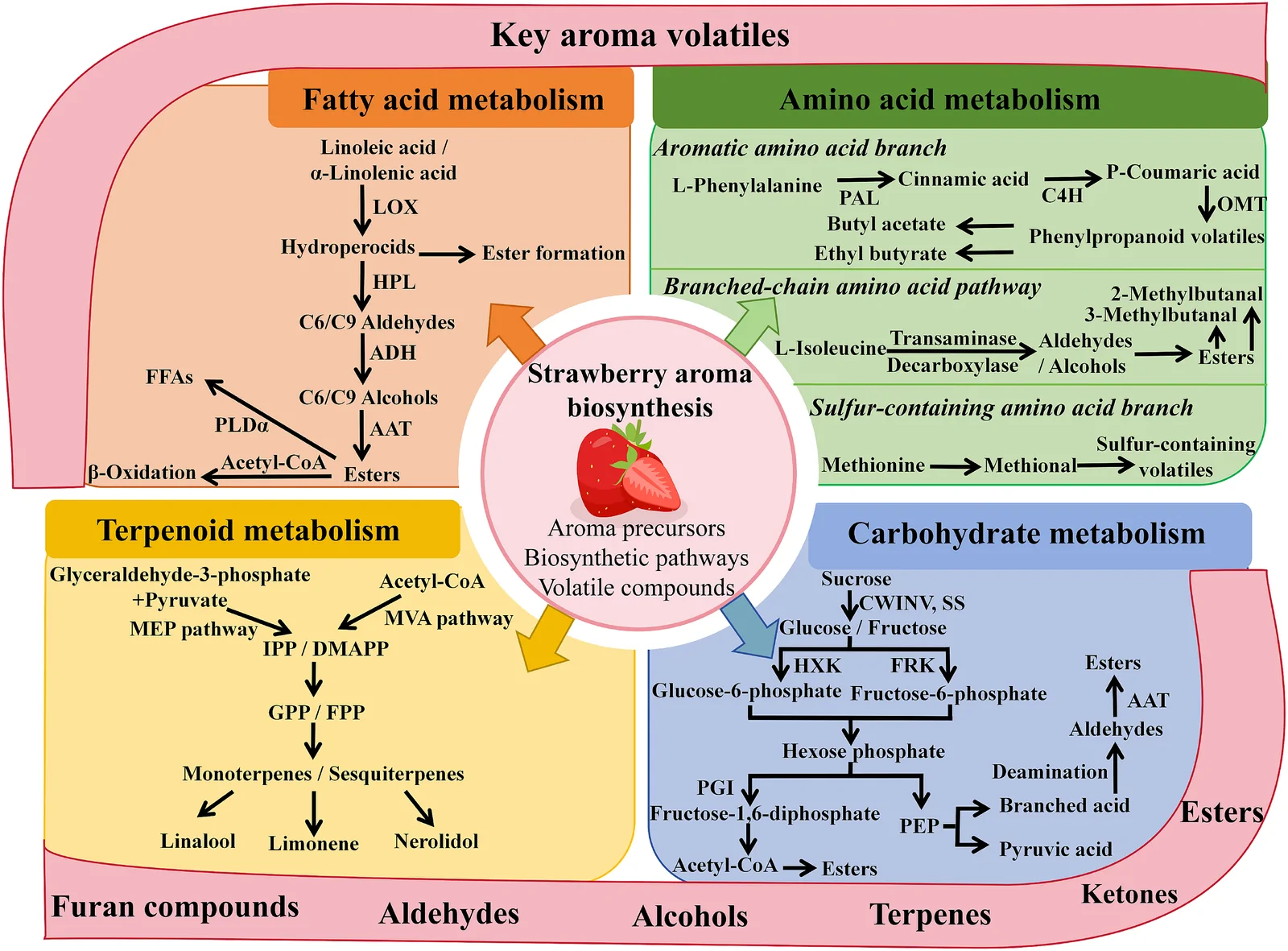
The metabolism pathways of key aroma volatiles in strawberries. LOX: Lipoxygenase pathway; HPL: Hydroperoxidase lyase; ADH: Alcohol dehydrogenase; AAT: Alcohol-acyltransferase; PLDα: Phospholipase D alpha; Acetyl-CoA: Acetyl coenzyme A; FFAs: Free fatty acids; PAL: Phenylalanine ammonia-lyase; C4H: Cinnamate-4-hydroxylase; OMT: *O*-methyltransferase; CWINV: Cell wall invertase; SS: Starch synthase; HXK: Hexokinase; FRK: Fructokinase; PGI: Phosphoglucose isomerase; PEP: Phosphoenolpyruvate; MEP: 2-*C*-methyl-D-erythritol 4-phosphate pathway; MVA: mevalonate pathway; IPP: Isoprene pyrophosphate; DMAPP: Dimethylallyl pyrophosphate; GPP: Geranyl pyrophosphate; FPP: Farnesyl pyrophosphate.

### 3.1 Fatty acid metabolism pathway

The formation of strawberry aroma compounds is primarily dependent on the metabolic pathways of fatty acids, amino acids, terpenoid, and carbohydrates, regulated by key enzymes such as alcohol acyltransferase (AAT). In the fatty acid metabolism pathway, volatile flavor compounds are predominantly produced via the LOX pathway and the *α*−/*β*-oxidation processes (Yan et al., 2018). Specifically, ester aroma components are derived from the catalytic reactions of C18 unsaturated fatty acids, such as linoleic acid and linolenic acid, within the LOX pathway. These polyunsaturated fatty acids are initially catalyzed by LOX, followed by HPL and isomerase, resulting in the production of C6 aldehyde compounds, including hexanal and (*E*)-2-hexenal. Subsequently, C6 aldehydes are converted into their corresponding C6 alcohols, such as 1-hexanol and (Z)-3-hexenol, through the reduction action of aldehyde dehydrogenase (ADH) (Lu et al., 2018). Building upon this, C6 alcohols further react with acyl-coenzyme A (acyl-CoA) generated from fatty acid *β*-oxidation, catalyzed by AAT, to form C6 esters like hexyl acetate and ethyl hexanoate (Lu et al., 2021). Consequently, this pathway constitutes a significant source of short-chain (six‑carbon) ester compounds. In addition to the LOX pathway, the *β*-oxidation process of fatty acids also provides precursors for long-chain esters, such as ethyl caproate, and lactone compounds, including *γ*-decanolactone. The synthesis of lactones originates from saturated fatty acids and involves multiple reactions, including dehydrogenation, epoxidation, hydration, hydroxylation, and *β*-oxidation. Ultimately, the key intermediate hydroxyacetyl-CoA undergoes internal esterification to produce the corresponding lactones (Peng et al., 2020). At the gene expression level, lipoxygenase and alcohol dehydrogenase genes are highly expressed during the early stages of fruit development, providing a substrate basis for the accumulation of alcohol substances. The expression level of the alcohol acyltransferase gene directly regulates the synthesis of esters, which affects the formation of the characteristic aroma of strawberries (Bian et al., 2023). Additionally, the transcription factor *FaMYB11* can positively regulate the activity of the LOX pathway during fruit ripening by directly binding to and activating the promoter of the *FaLOX5* gene, thereby significantly promoting the accumulation of volatile esters such as 3-methylbutanoate methyl ester and enhancing the aroma characteristics of strawberries (Lu et al., 2021).

### 3.2 Amino acid metabolism pathway

The generation of plant aroma compounds is closely linked to amino acid metabolism, with the phenylalanine pathway and branched-chain amino acid pathway serving as the two primary metabolic sources (Maoz et al., 2022). In the phenylalanine pathway, phenylalanine is converted into phenolic volatile substances, including phenylacetaldehyde and phenylethanol, through a series of enzymatic reactions, thereby imparting floral characteristics to strawberries. Conversely, leucine, isoleucine, and valine from branched-chain amino acids undergo transamination and decarboxylation, leading to the formation of corresponding branched aldehydes. These aldehydes can then be reduced to branched alcohols by ADH. Subsequently, under the catalysis of AAT, branched alcohols react with acyl-CoA to produce branched esters with rich fruity aromas, such as ethyl 2-methylbutyrate, ethyl 3-methylbutyrate, and isoamyl acetate (Mostafa et al., 2022). Amino acid metabolism not only provides precursor substances for ester biosynthesis but also facilitates the activation of 2-oxoacid decarboxylase/dehydrogenase during its metabolic processes, promoting the formation of aromatic compounds such as alcohols, aldehydes, and acids (Akbari et al., 2025; Wu et al., 2024). In strawberry fruit, the regulation of amino acid metabolism is particularly crucial. For instance, the high expression of the *FaPAL1* and *FaDAHPS* genes during the turning red stage drives the accumulation of trans-cinnamic acid, while the increased expression of *FaCNL* further enhances the content of benzene metabolites such as methyl salicylate, phenylethanol, and benzaldehyde, leading to a significant accumulation of these aroma components (Bian et al., 2023). In the phenylpropanol biosynthesis pathway, phenylalanine is first deaminated by PAL to form cinnamoyl-CoA, which is subsequently converted to p-coumaroyl-CoA (Dong & Lin, 2021). Additionally, phenylalanine and tyrosine can be deaminated to produce *p*-coumaric acid, which is ultimately converted into intermediate compounds such as caffeoyl-CoA, feruloyl-CoA, and *p*-coumaroyl-CoA by 4-coumaric-CoA ligase. These compounds are further metabolized to generate aroma components such as 4-hydroxybenzaldehyde and phenolic esters (Fang et al., 2024).

### 3.3 Carbohydrate metabolism pathway

The role of carbohydrate metabolism in the aroma of strawberry fruits extends beyond the traditional perception of merely providing “sweetness.” Soluble sugars in the fruit undergo primary metabolic processes, including glycolysis, the pentose phosphate pathway, and the tricarboxylic acid cycle. These processes generate key intermediates such as acetyl coenzyme A (Acetyl-CoA), pyruvic acid, and phosphoenolpyruvic acid. Subsequently, these metabolic intermediates enter various pathways: the fatty acid pathway, amino acid pathway, terpene pathway, and furanone pathway. This entry provides carbon skeleton precursors essential for the biosynthesis of diverse aroma components, including esters, lactones, monoterpenes, and furanones (Baldi et al., 2018).

In the synthesis of furanone compounds characterized by a strawberry aroma, the relationship between carbohydrate precursors and aromatic substances has been confirmed through isotope tracing experiments. By applying ^13^C and ^2^H-labeled d-glucose and d-fructose to isolated strawberry fruits and utilizing gas chromatography–mass spectrometry (GC–MS) to trace the synthesis pathways of labeled atoms in DMHF and its methylated derivative DMMF, it is established that d-fructose-6-phosphate serves as the direct precursor for the carbon skeleton of furanones. Additionally, d-glucose must be isomerized to d-fructose-6-phosphate by phosphoglucoisomerase before entering this metabolic pathway (M et al., 2001). Based on this, the FaEO/FaQR furanone oxidoreductase was identified as the key enzyme catalyzing the terminal oxidation reaction of this pathway. Additionally, the FaOMT1 further methylates DMHF to DMMF, thereby completing the metabolic conversion chain from carbohydrates to furanone aroma compounds (Badovskaya et al., 2021).

The regulation of aroma compounds by carbohydrates extends beyond biosynthesis; it also plays a crucial role in the dynamic equilibrium of aroma storage and release. UDP-glycosyltransferases UGT71K3a/b and UGT85K16 utilize UDP-glucose as the sugar donor to glycosylate volatile free-state aroma compounds, such as DMHF, into *β*-D-glucosides, subsequently storing them in vacuoles (Sheng et al., 2025). Gene silencing of *UGT71K3* results in a significant reduction in the accumulation of HDMF-sugars. Additionally, the decline of free-state HDMF in the fruit during the late stage of ripening is markedly slowed, indicating that glycosylation modification serves a “dynamic storage “ physiological function for aroma compounds (Song et al., 2016). In contrast, the glycoside hydrolase family members FaBGLU40 and FaBGLU42 can be transcriptionally upregulated in response to environmental signals, such as low light and low temperature. These enzymes catalyze the cleavage of glycosidic bonds, thereby converting flavorless glycoside-bound aroma compounds back into volatile, free-state compounds that possess sensory activity (Sheng et al., 2025). The reversible regulatory loop that senses the activation of glycosidase by environmental factors and facilitates the release of aroma offers a novel perspective for understanding the molecular mechanisms underlying aroma compensation during the post-harvest storage of strawberries (Chen & Quek, 2023).

The Acetyl-CoA produced from carbohydrate metabolism enters the fatty acid oxidation pathway. Following peroxidation and cleavage reactions catalyzed by lipoxygenase, C6 and C9 aldehyde compounds are generated. These compounds are subsequently converted into fruit aroma esters, such as ethyl hexanoate and ethyl butanoate, through the synergistic action of alcohol dehydrogenase and alcohol acyltransferase (Rey-Serra et al., 2022). It is important to note that the fatty acid pathway does not operate in isolation. QTL mapping and candidate gene function verification indicate that the enhancement of *γ*-decalactone biosynthesis may coincide with a reduction in the levels of certain sugars and free amino acids in the fruit. This observation suggests a competitive redistribution relationship between carbohydrate metabolism and aroma components, which is regulated in a coordinated manner by multiple enzymatic nodes and transcription factors (Fan et al., 2021). In summary, the interaction between carbohydrate metabolism and strawberry aroma is primarily evident in three functional aspects: precursor supply, reversible storage through glycosylation, and cross-pathway distribution. The molecular regulatory network encompasses the expression of sugar transport proteins, UDP-glycosyltransferases, glycoside hydrolases, and several key enzymes involved in secondary metabolic pathways.

### 3.4 Terpenoid metabolism pathway

The biosynthesis of terpenoids primarily occurs through two pathways: the mevalonate (MVA) pathway and the 2-*C*-methyl-D-erythritol 4-phosphate (MEP) pathway (Liu et al., 2023; Madera et al., 2026; Urrutia et al., 2017; Zheng et al., 2021). In the MVA pathway, glucose is metabolized via glycolysis to produce acetyl-CoA, which is then condensed with three molecules of acetyl-CoA to form mevalonic acid. Mevalonic acid undergoes a series of phosphorylation and decarboxylation reactions, ultimately yielding isopentenyl pyrophosphate (IPP) and dimethylallyl pyrophosphate (DMAPP). The MEP pathway, occurring in cytoplasmic bodies, utilizes glyceraldehyde-3-phosphate and pyruvic acid as precursors, leading to the production of IPP and DMAPP. Subsequently, under the catalytic action of isoprenoid transferase, IPP condenses with DMAPP to form chain precursors, including geranyl pyrophosphate as a monoterpene precursor and farnesyl pyrophosphate (FPP) as a sesquiterpene precursor. These precursors are further catalyzed by TPS for cyclization or modification, ultimately resulting in compounds such as the monoterpene linalool and the sesquiterpene myrtenol (Liu et al., 2023; Madera et al., 2026; Urrutia et al., 2017). During the development of strawberry fruit, key genes involved in various synthetic pathways exhibit stage-specific expression. For instance, the *FaNES1* gene is highly expressed in the MVA pathway during the red stage of fruit development, promoting an increase in the content of the sesquiterpene derivative naringenin. Concurrently, multiple genes in the MEP pathway, such as *FaDXR*, are significantly upregulated during the color transition phase, which subsequently influences the synthesis of monoterpenes (Bian et al., 2023). Additionally, during the fruit ripening period, the expression of the key gene 4-hydroxy-3-methyl-2-enyl diphosphate reductase in the MEP pathway shows a moderate increase, while the transcriptional abundance of the rate-limiting enzyme 3-hydroxy-3-methylglutaryl-CoA reductase also significantly rises. Notably, the high expression of *FaTPS50* in white fruit is associated with the accumulation of immature *α*-pinene (Madera et al., 2026).

## 4 Factors affecting strawberry aroma

The aroma of strawberries is primarily determined by their aromatic active compounds, which, in combination with their categories and concentrations, create a unique scent. Factors such as genotype or variety, cultivation conditions, fruit developmental stage, and postharvest handling significantly influence the expression of strawberry aroma (Fig. 3).

**Fig. 3:**
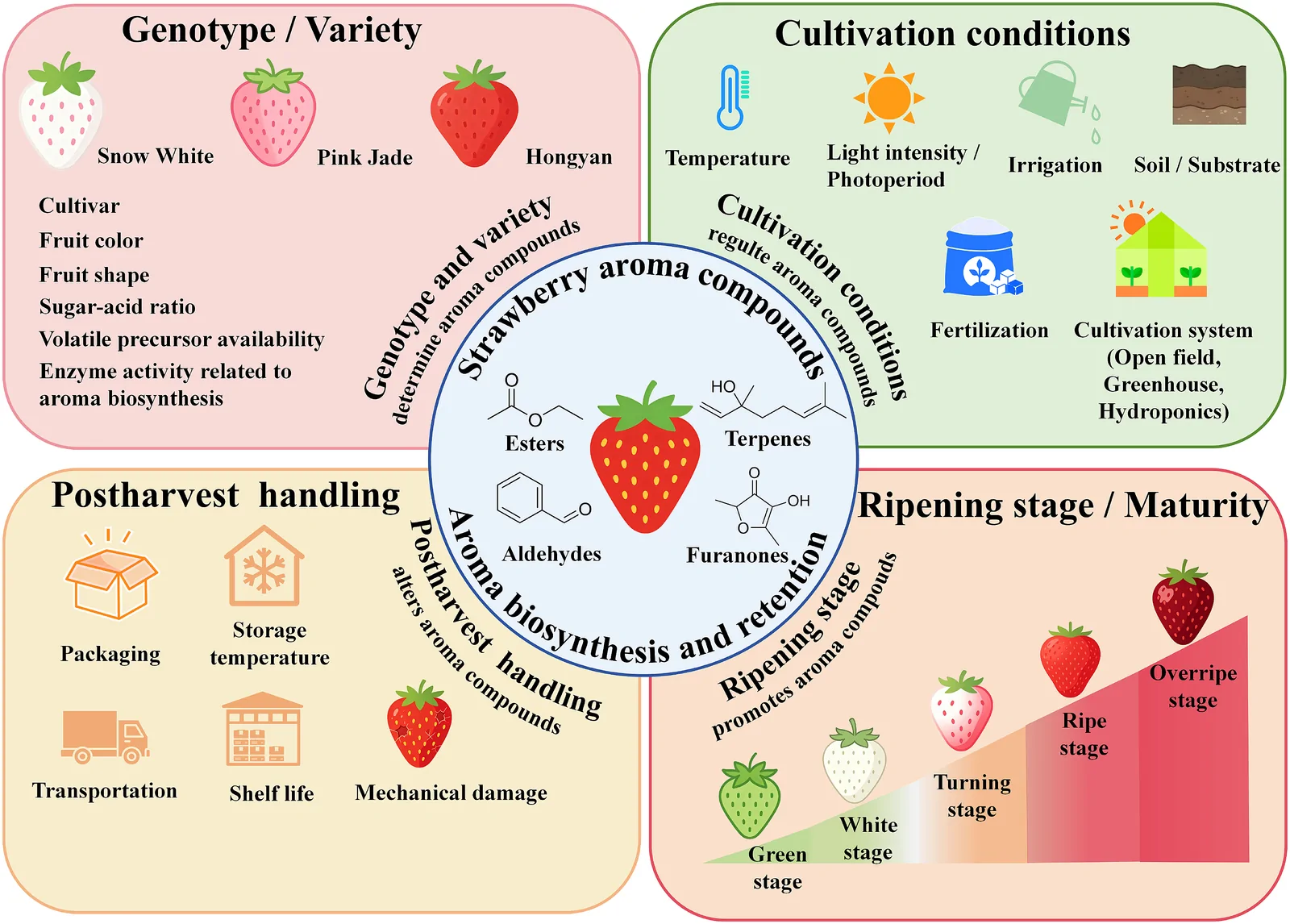
The factors affecting the generation of aroma compounds in strawberries.

### 4.1 Genotypes or varieties

Strawberries, widely cultivated as economic crops globally, exhibit a rich diversity of varieties and aroma characteristics. The American variety “Sweet Charlie” is primarily noted for its fresh fruity aroma, large fruit size, and strong disease resistance, contributing to its widespread cultivation. In contrast, the Japanese variety “Hongyan” is celebrated for its rich rose aroma, while another representative Japanese variety, “Zhangji,” is referred to as the “cream strawberry” due to its distinctive creamy scent (Scott et al., 2021; Sheng et al., 2021; Zhang et al., 2025). Domestically bred varieties also showcase unique aromatic profiles: “Pink Jade” features a compound aroma reminiscent of lychee and honey, “Jing Tao Xiang” offers a fragrance akin to yellow peach, and “Snow White” presents tropical fruit aromas such as pineapple and coconut (Xu, Shi, et al., 2024). These aromatic differences primarily arise from variations in the types and concentrations of VOCs across different varieties. VOCs include esters, aldehydes, alcohols, ketones, and terpenes, which collectively contribute to the aroma characteristics of strawberries. The analysis conducted by Sheng et al. (2021) on 16 strawberry varieties revealed that esters were the predominant compounds across all varieties, followed by alcohols and aldehydes, while ketones exhibited the lowest content. Through aroma value analysis, a total of 16 characteristic aroma components were identified, indicating that the composition and content of VOCs directly influenced the type and intensity of aroma (Sheng et al., 2021). Sheng et al. (2025) systematically classified the aromas of six major strawberry varieties cultivated in China, categorizing “Hongyan” as fruity aroma, “Xiangye,” “Xuexiang,” and “Snow White” as floral aroma, “Taoxundai” as peach blossom aroma, and “Christmas Red” as pineapple aroma (Sheng et al., 2025). The differences in aroma characteristics are primarily regulated by the genetic background of each variety. The accumulation levels of specific key aroma compounds are determined by genotype; for instance, the contents of furanone (DMMF) and gamma-decanolactone are entirely governed by genetic factors, thereby endowing varieties with unique sweet or fruity aromas (Leonardou et al., 2021). For example, the relative concentration of gamma-decanolactone in “Jing Tao Xiang” is significantly higher than in other varieties, contributing to its distinctive peach fragrance. Similarly, the rich sweet aroma of “Rose Princess” stems from its genotype, which is characterized by elevated levels of DMMF and agarwood alcohol expression. This analysis underscores that the genetic regulation of aroma metabolism not only accounts for the aromatic differences among varieties but also enriches the genetic diversity of strawberries.

### 4.2 Cultivation conditions

The flavor quality of strawberries is significantly influenced by various environmental and cultivation factors. Research indicates that different soil types, altitude conditions, and cultivation management practices result in substantial differences in the composition and concentration of aroma compounds in strawberries, thereby affecting their overall aroma characteristics. A comparison of strawberries cultivated in spring and winter revealed that the total amount of volatile compounds in spring fruits was generally lower than that in winter fruits, with a notable decrease in alcohols, esters, alkanes, and alkenes. Interestingly, 4-oxo-hex-2-enal exhibited a higher concentration in spring fruits, and this seasonal variation in aroma composition was primarily attributed to temperature fluctuations in the growth environment. Warm conditions may inhibit the biosynthesis or accumulation of key aroma compounds (Do et al., 2024). In addition to seasonal effects, cultivation methods also play a crucial role in determining aroma quality. According to Xu et al. (Xu, Liu, et al., 2025), substrate cultivation can optimize the oxygen and water conditions in the rhizosphere of “red-skinned” strawberries compared to traditional soil cultivation, thereby increasing the content of esters such as ethyl acetate and certain alcohols (Xu, Shi, et al., 2025). However, this effect may vary depending on the variety. Li et al. (2021) observed that soil cultivation significantly enhances the accumulation of key aroma components, such as methyl butyrate, ethyl caproate, HDMF, and DMMF, in “Amaou” strawberries. It is speculated that substrate cultivation may restrict aroma synthesis capacity to some extent due to variations in water availability, nutrient levels, or microbial communities (Li, Zhang, et al., 2021). Additionally, altitude plays a crucial role in regulating aroma composition by influencing the accumulation of photosynthetic products and secondary metabolites. Reports indicate that strawberries cultivated at high altitudes typically exhibit higher sweetness and richer flavors compared to their low-altitude counterparts, which are often dominated by perilla alcohol. This suggests that light and temperature conditions, along with the metabolic background, significantly impact the distribution of aroma compounds (He et al., 2025). Furthermore, cultivation management practices, including the type and application method of fertilizers, also affect aroma metabolism pathways. Different organic fertilizer treatments can modify the levels of key metabolites such as phenylalanine, leucine, and salicylic acid, leading to variations in flavor (Liu et al., 2024). Jatana et al. (2025) further elucidated that the forms of nitrogen fertilizers, specifically ammonia nitrogen and organic nitrogen, can regulate the tricarboxylic acid cycle and amino acid metabolism. This regulation subsequently affects the supply of aroma precursors, such as acetyl-CoA, and modulates the synthesis of volatile components, including esters and alcohols (Jatana et al., 2025).Additionally, Duan et al. (2021) compared the effects of root application and foliar application of yeast fertilizer. Their findings indicate that root application may enhance photosynthesis and sugar accumulation, thereby promoting the formation of aroma compounds such as DMMF (Duan et al., 2021).

In summary, soil type significantly influences nutrient availability, particularly concerning nitrogen, sulfur, and micronutrients, which act as precursors or cofactors in the biosynthesis of esters, sulfur-containing volatiles, and terpenoids. Altitude conditions, including temperature, light intensity, and diurnal temperature variation, modulate the photosynthetic carbon supply and the expression of genes involved in the lipoxygenase, terpenoid, and phenylpropanoid pathways. Cultivation management practices, such as fertilization, irrigation, pruning, and mulching, modify plant water status, hormone signaling, and secondary metabolism, thereby impacting the accumulation of aroma-active compounds. These effects are often interactive and genotype-dependent, necessitating further investigation into their mechanistic basis.

### 4.3 Ripening stages of strawberry fruit

The complete development process of strawberry fruit, from flowering to maturity, can be divided into five distinct stages: the small green fruit stage (15 days after flowering), the large green fruit stage (20 days after flowering), the white fruit stage (30 days after flowering), the pink fruit stage (35 days after flowering), and the full red fruit stage (40 days after flowering). At each stage of maturity, the composition and concentration of flavor compounds in strawberry fruits undergo significant changes, ultimately shaping their final flavor characteristics (Padilla-Jiménez et al., 2021). During the green fruit stage, aldehydes predominate, constituting 70% of the total volatile compounds, with (*E*)-2-hexenal and hexanal serving as key representatives, imparting a distinct grassy aroma to the fruit (Zheng et al., 2023). Concurrently, phenolic substances accumulate in substantial quantities, with flavonoids and hydroxybenzoic acid derivatives making up 87–95% of the total phenolic content (Aubert et al., 2021). As the fruit transitions to the pink fruit stage, the aldehyde content decreases by 24–49%, while the levels of esters (such as ethyl acetate and methyl hexanoate) and alcohols (such as linalool) significantly increase, leading to the development of typical fruity aromas (Li, Brouwer, et al., 2021; Simkova et al., 2024). This transformation is closely associated with the heightened activity of LOX and AAT, which together catalyze the conversion of fatty acids into esters, including hexyl acetate (Parra-Palma et al., 2019). At the fully red fruit stage, the types and contents of esters reach their peak, with representative components including ethyl butyrate, ethyl caproate, and *γ*-decanolactone, accounting for 23–64% of the total volatile matter. The types of aroma substances increase approximately 14-fold compared to the immature stage, with ethyl caproate, (*E*)-2-hexenal, and DMMF emerging as key contributors to the characteristic flavor of strawberries due to their low aroma threshold and high aroma activity value. At this stage, both AAT and ADH activities peak, with ADH activity showing a significant positive correlation with the synthesis of ethyl butyrate, thereby jointly promoting the final formation of ester aroma compounds. Different strawberry varieties also exhibit metabolic differences at the same maturity stage, which in turn affect their flavor characteristics. For instance, during the color transition period, the content changes of substances such as quinic acid and 2-methylmaleic acid in the amino acid metabolism pathway of “Oriental Snow” and “Oriental Beauty” show opposite trends, resulting in significantly different aroma profiles between the two (Fang et al., 2024). Furthermore, volatile fatty acids such as methyl butyrate and methyl caproate accumulate with increasing maturity, imparting a floral and fruity aroma to mature strawberries (Li, Brouwer, et al., 2021). In the “Sweet Charlie” strawberry, the immature stage is primarily characterized by herbaceous substances such as octylcyclohexane, while the concentration of terpenes peaks during initial maturity, contributing to a floral aroma. At the fully mature stage, esters represented by methyl hexanoate dominate (Padilla-Jiménez et al., 2021). The differences between these varieties further indicate that the formation of strawberry flavor is not only dependent on shared metabolic pathways but is also regulated by genetic background.

### 4.4 Postharvest storage

The storage process of strawberries, from harvesting to consumption, is crucial for maintaining their quality, as changes in aroma components directly influence consumer acceptance. During storage, volatile compounds such as methyl caproate, linalool, and furanone in strawberries undergo dynamic changes through metabolic pathways, reshaping the aroma profile. Research indicates that with prolonged storage time, the ester compound ethyl acetate accumulates via fatty acid and amino acid metabolic pathways, enhancing fruity characteristics. However, extended storage can lead to an increased concentration of certain alcohols, such as 1-propanol and 3-octanol, resulting in quality deterioration (Di et al., 2022). Additionally, storage temperature significantly influences aroma evolution. Heat treatment promotes the synthesis of overall aroma components, while cold treatment reduces the proportion of linalool, thereby weakening the floral aroma (Zheng et al., 2022). Furthermore, enzyme activity is critical in the refrigeration process. A decrease in the activity of key ester biosynthesis enzymes results in a reduced proportion of non-acetate substances. Simultaneously, the activities of FPP synthase, linalool synthase, and peroxidase related to the phenylpropanol pathway are diminished, collectively altering the composition of VOCs (Baldwin et al., 2023). Besides storage time and temperature, preservation treatments and packaging methods also significantly impact aroma retention. Ultrasonic pretreatment can enhance the content of key compounds such as linalool and ethyl caproate, producing “apple” and “green leaf” flavors, thus maintaining aroma quality (Li et al., 2024; Xu, Quan, et al., 2024). Chitosan preservatives delay fruit aging and preserve flavor by promoting the accumulation of methyl caproate during long-term refrigeration (Georgiadou et al., 2025).

## 5 The analysis and recognition technologies of strawberry aromas

Fig. 4 illustrates the integration of analytical and recognition technologies, encompassing sensory and pattern recognition, electronic sensing, and chromatography-based instrumental analysis, alongside multi-omics approaches including genomics, transcriptomics, proteomics, and metabonomics, in the examination of volatile aroma compounds in strawberries.

**Fig. 4:**
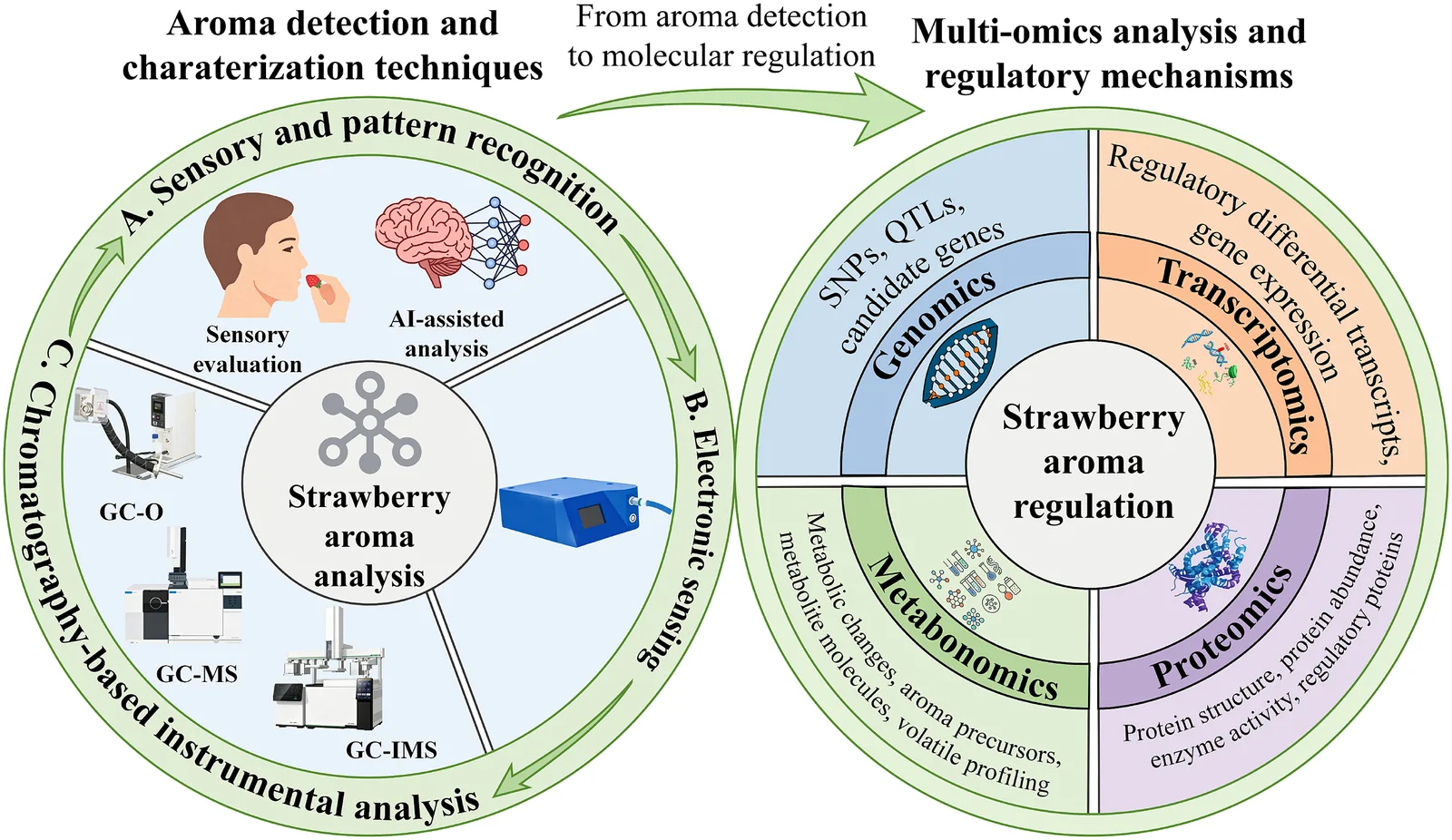
The integration of analysis and recognition technologies with multi-omics approaches in the analysis of volatile aroma compounds in strawberries. GC-O: Gas Chromatography-Olfactometry; GC–MS: Gas Chromatography-Mass Spectrometry; GC-IMS: Gas Chromatography-Ion Mobility Spectrometry; SNPs: Single Nucleotide Polymorphisms; QTLs: Quantitative Trait Locus.

### 5.1 Sensory evaluation

Sensory evaluation, the most prevalent analytical method for identifying the sensory characteristics of food and measuring consumer preferences, focuses on complex sensory attributes such as aroma, which is primarily perceived through smell and is simultaneously influenced by taste and synergistic effects from other senses. During oral processing and nasal inhalation, volatile components are detected by olfactory epithelial receptors and transmitted to the brain for neural integration, ultimately resulting in aroma perception (Zhan et al., 2020). Aroma is a critical factor in determining the sensory quality of food and significantly influences consumers' purchasing desires. In the evaluation of strawberry quality, a sensory quality index based on consumer perception has been constructed, enabling scientific judgment on strawberry quality control and shelf life through systematic evaluations conducted by professional reviewers (Tomadoni et al., 2018). Sensory evaluation typically falls into two categories: analytical and consumer. Analytical evaluation primarily employs descriptive analysis, where the sensory attributes of products are quantitatively assessed by trained evaluators (Barbe et al., 2021). Moazzem et al. (2024) utilized descriptive sensory evaluation to identify typical odor characteristics in strawberries, such as “fruity,” “floral,” “sweet,” and “sour” (Moazzem et al., 2024). Xiaofen et al. (2011) revealed the presence of complex aroma profiles, including “floral aroma,” “grassy aroma,” “sweet aroma,” “fruity aroma,” and “volatile sour aroma” in two subtropical strawberry varieties through sensory description analysis. Consumer sensory evaluation focuses on assessing the overall or individual attributes (such as appearance, smell, and taste) of a product based on consumer preferences, thereby reflecting market acceptance directly (Xiaofen et al., 2011). Although olfactory evaluation of strawberry aroma offers intuitive and real-time insights, this method is inherently subjective due to olfactory fatigue and individual perceptual differences, which can result in significant errors and insufficient repeatability in the findings. Therefore, to enhance the accuracy and reliability of evaluation results, it is essential to provide professional training to reviewers and integrate modern instrumental analysis techniques for the precise and efficient identification of aroma compounds.

### 5.2 E-nose technology

The perception of flavor has evolved from a reliance on subjective sensory experiences in earlier times to the utilization of modern instruments for precise analysis, thereby facilitating a deeper understanding of aroma formation mechanisms and the processes involved in perception. Given that reliance on human olfactory senses is prone to fatigue and subjective bias, research has introduced electronic nose (E-nose) technology to objectively identify categories of volatile aroma components. This sensor system can stably and repeatedly detect substances, effectively compensating for the limitations of sensory evaluation and demonstrating unique advantages in identifying aroma components that are challenging for humans to perceive (Cheng et al., 2021; Moazzem et al., 2024). E-nose technology integrates multiple sensors and electronic aroma detection methods to achieve the recognition and classification of complex aroma mixtures. For instance, Xing et al. (2018) developed a novel E-nose device that captured volatile odor signals released during the storage and transportation of strawberries and constructed a data analysis model to effectively differentiate between spoiled and fresh fruits. The reliability of the results was validated through GC–MS (Xing et al., 2018). Rao et al. (2020) employed an E-nose to detect changes in volatile compounds induced by vibration damage in strawberries, aiming to predict the extent of fruit damage (Rao et al., 2020). Through E-nose detection and analysis, the differences in aroma characteristics among various strawberry varieties under identical cultivation conditions were elucidated, thereby providing a foundation for variety identification (Xu, Shi, et al., 2024). Furthermore, advancements have been made in the application of E-nose technology in strawberry processing. By monitoring the overall evolution of aroma in strawberry juice during storage and simulating microbial changes, it is determined that the increase in total bacterial count is closely associated with the process of aroma deterioration (Zhang et al., 2022). In addition, compared to traditional sensory evaluation methods, E-nose demonstrates greater efficiency in analyzing the aroma of strawberry juice, thus offering a novel approach for qualitative identification and quantitative prediction of quality parameters (Qiu et al., 2015).

### 5.3 GC–MS, GC-IMS, GC-O technologies

Although sensory evaluation and E-nose technology can efficiently characterize the macroscopic profile of strawberry aroma, they are limited by subjective judgment bias and face challenges in accurately identifying and structurally characterizing volatile components. Consequently, HS-SPME-GC–MS, gas chromatography ion mobility spectrometry (GC-IMS), and GC-O techniques are increasingly playing a crucial role in the study of strawberry aroma. HS-SPME-GC–MS is widely employed for the qualitative and quantitative analysis of volatile components in strawberries, owing to its solvent-free extraction method, high sensitivity, and advantages in multi-component separation. In a comparative analysis of freshly squeezed juices from three strawberry varieties—”Miaoxiang 3,” “Hongyan,” and “Qianmei 2”—HS-SPME-GC–MS identified a total of 89 volatile compounds, including esters, aldehydes, alcohols, ketones, and acids, revealing compositional differences among the varieties. For instance, “Hongyan” exhibited higher concentrations of hexyl acetate and (*Z*)-acetoxy-2-hexen-1-ol ester, whereas “Qianmei 2″ predominantly contained hexanal and (*E*)-2-hexenal (Zhang et al., 2023). GC-IMS is advantageous for the rapid identification and comprehensive flavor profile analysis of strawberry samples, attributed to its high sensitivity, swift analysis, and straightforward sample pretreatment characteristics. By integrating visual fingerprint maps generated by GC-IMS with chemometric techniques, distinct strawberry varieties were effectively identified, successfully differentiating between “Hongyan,” “Zhangji,” and “Snow White,” while screening 26 potential biomarkers for rapid classification (Xu, Shi, et al., 2024). Conversely, GC-O technology identifies active compounds significantly contributing to strawberry aroma by merging instrumental analysis with sensory evaluation, often supplemented by OAV for comprehensive analysis. In investigation of heat-sterilized strawberry juice, GC-O combined with OAV identified dimethyl trisulfide (associated with a boiling cabbage flavor), (*E*)-3-hexenol (which imparts a medicinal flavor), and hexanoic acid (contributing to a roasted fruit flavor) as key odorants. Furthermore, it was observed that a reduction in ethyl hexanoate and (*E*)-2-octenal led to a diminished fruit aroma (Yao et al., 2025). A high OAV does not necessarily equate to sensory contribution due to compound interactions and matrix effects in strawberry fruit.

To better clarify the selection of methods for aroma analysis, the advantages and limitations of these three techniques are compared below. HS-SPME-GC–MS offers strong separation capabilities, high sensitivity, and reliable qualitative and quantitative analyses of a wide range of volatile compounds, making it particularly suitable for compound identification and quantification. However, this method is relatively time-consuming, and its extraction efficiency can be influenced by fiber coating selectivity, sample matrix, and volatilization behavior, which may result in the underestimation or overestimation of certain compounds. GC-IMS is rapid, sensitive, and requires minimal sample pretreatment; its visual fingerprint maps are well-suited for the rapid discrimination and classification of strawberry varieties. Nevertheless, its qualitative and quantitative capabilities are generally weaker than those of GC–MS, and accurate identification often relies on database matching and chemometric modeling (Xu, Shi, et al., 2024). GC-O uniquely combines chromatographic separation with human olfactory detection, making it an indispensable technique for identifying key odor-active compounds and evaluating their sensory contributions, especially when combined with OAV (Mahmoud & Zhang, 2024). However, this method is subjective, labor-intensive, and time-consuming, and it typically needs to be coupled with GC–MS to confirm the structures and concentrations of the detected odorants. Therefore, these three techniques should be regarded as complementary, and their combined use can provide a more comprehensive and reliable understanding of strawberry aroma.

### 5.4 Genomics technology

The in-depth study of strawberry genomics has laid a solid foundation for analyzing the mechanisms underlying aroma formation. This research not only enhances our understanding of the biological characteristics of strawberries—including growth cycles, fruit development, and disease resistance—but also provides crucial theoretical support for genetic improvement and variety innovation (Hardigan et al., 2018; Pincot et al., 2020). The application of whole genome sequencing technology has unveiled the complex structure and diversity of the strawberry genome, thereby facilitating subsequent gene function analyses and breeding efforts (Kang et al., 2013). By employing *CRISPR-Cas9* gene editing techniques to precisely modify the specific gene *FvebZIPs1.1*, a significant increase in fruit sugar content was achieved. Multiple strains devoid of exogenous genes were produced through self-fertilization and hybridization, thereby opening new avenues for regulating flavor quality (Xing et al., 2020). Additionally, Qiao et al. (2021) utilized high-throughput sequencing technology to systematically analyze the genomic data of various wild strawberries and key species, providing a foundational basis for elucidating the gene expression regulatory network during strawberry growth and development (Qiao et al., 2021). In terms of aroma component analysis, research has confirmed that the lactone compound gamma-decalactone significantly contributes to the distinctive peach fragrance of strawberries. Metabolite profiling, combined with gene function studies, has identified *FaFAD1* as a pivotal factor regulating the synthesis of this lactone. Furthermore, investigations into sugar accumulation-related genes have revealed multiple differentially expressed genes involved in the metabolism of fructose, glucose, and sucrose. These genes serve as key regulatory elements for the production of gamma-decalactone in “Zhangji” strawberries (Oh et al., 2021). By analyzing sugar content-related genes in strawberries, genes associated with the regulation of fructose, glucose, sucrose, and soluble sugars have been identified, including *FaFru3*, *FaSuc11*, and *FaGlu8*. The expression patterns of these genes are closely linked to sugar variations among strawberry varieties, thereby indirectly influencing aroma perception (Ashokraj et al., 2017). Additionally, through metabolic engineering, the combined inhibition of chalcone synthase genes and the overexpression of eugenol or isoeugenol synthase can redirect flavonoid metabolism towards the synthesis of phenylpropanoids, enabling cultivated strawberries to preserve the aromatic characteristics of their wild counterparts (Hoffmann et al., 2011).

### 5.5 Transcriptomics technology

Transcriptomics serves as a valuable tool for analyzing the molecular mechanisms underlying strawberry aroma formation. Through a systematic examination of the dynamic changes in gene expression throughout fruit ripening, several key genes that regulate the synthesis of sugar, acid components, and volatile aroma compounds have been successfully identified. Chambers et al. (2014) identified the *FaFAD1* gene by integrating phenotypic identification and transcriptomic methods, which directly regulated the synthesis of volatile compounds that were core flavor components of strawberries (Chambers et al., 2014). Lu et al. (2021) discovered that *FaMYB11*, functioning as a transcription factor, regulated the *FaLOX5* gene within the LOX pathway, thereby promoting the accumulation of volatile compounds derived from fatty acids during strawberry ripening. Concurrently, an in-depth analysis of ester metabolism pathways revealed that genes such as *FaCXE2* and *FaCXE3* negatively regulated ester accumulation, directly influencing the intensity and composition of strawberry aroma (Lu et al., 2021). Regarding terpene aroma, it is established that the *FanNES1* gene plays a crucial role in terpene synthesis, enhancing the terpene aroma characteristics of strawberries by regulating the content of compounds like linalool (Barbey et al., 2021). Furthermore, the transcriptional inhibitory factor *FaMADS1* has been found to negatively regulate multiple maturity-related genes. Under refrigeration conditions, the downregulation of *FaTRAB1* alleviates the inhibition of *FaMADS1* through epigenetic mechanisms, synergizing with the expression changes of genes such as ADH, which leads to a reduction in ester biosynthesis pathways and ultimately affects aroma expression (Baldwin et al., 2025). Recent studies have also indicated that external treatments, such as ultrasound, can upregulate the *FaNES1* gene, promoting the synthesis of terpenoid compounds in accordance with the observed trends in aroma changes (Li et al., 2024). Through comparative transcriptome analysis, the *FanAAMT* methyltransferase is identified, which catalyzes the biosynthesis of MA, further elucidating the formation pathway of specific aroma compounds (Pillet et al., 2017).

### 5.6 Proteomics technology

Proteomics systematically identifies key enzyme proteins involved in the synthesis of volatile aroma compounds and analyzes their regulatory networks, revealing the molecular mechanisms underlying aroma formation. This includes dynamic changes in metabolic pathways, environmental response mechanisms, and genetic differences between varieties. Lu et al. (2020) studied that the expression level of the *FaLOX5* gene increased synchronously with protein expression during strawberry fruit development, indicating its important function in the LOX pathway. Meanwhile, the transcription factor *FaMYB9* has been confirmed to regulate the biosynthesis of C6 volatile compounds. Overexpression experiments demonstrated a positive correlation between changes in C6 substance content and LOX pathway activity, further elucidating the key regulatory nodes of aroma synthesis (Lu et al., 2020). Additionally, proteomics is often integrated with metabolomics and transcriptomics to systematically identify differentially expressed proteins (such as enzymes), metabolites (including volatile components like esters and terpenes), and gene expression profiles under strawberry development or environmental stress, thereby constructing a comprehensive regulatory network for aroma synthesis.

### 5.7 Metabolomics technology

In the study of the mechanisms underlying strawberry flavor formation, metabolites act as precursors or intermediates of flavor compounds. The variations in their types and quantities directly influence the synthesis and accumulation of these flavor compounds (Zhu et al., 2019). Consequently, metabolomics is extensively employed to identify key metabolites and associated metabolic pathways, thereby elucidating the biological basis of aroma formation. Metabolomics is typically categorized into two main types: non-targeted and targeted approaches. Non-targeted methods, such as liquid chromatography-mass spectrometry and GC–MS, are adept at broadly screening metabolites. In contrast, targeted methods quantitatively analyze specific metabolites to validate potential biomarkers. In the investigation of strawberry aroma, HS-SPM*E*-GC–MS has emerged as a widely utilized technique for analyzing volatile compounds. Zhang et al. (2023) employed HS-SPME-GC–MS and GC-IMS to analyze strawberry juice and discovered that key aroma components, including hexyl caproate, ethyl 2-methylbutyrate, and ethyl octanoate, significantly decreased in concentration during refrigeration, resulting in a deterioration of aroma quality. Furthermore, this method effectively distinguishes samples from different varieties and storage durations (Zhang et al., 2023). Song et al. (2023) identified 131 flavor compounds in strawberries using targeted metabolomics with GC–MS technology and reported, for the first time, the presence of ethyl vanillin, which was characterized by its “vanilla flavor,” thereby enhancing the understanding of strawberry aroma diversity (Song et al., 2023). A comprehensive metabolomics analysis of ten strawberry varieties conducted by Wang et al. (2023) revealed significant differences in metabolites among varieties and indicated that amino acid metabolism, flavonoid synthesis, and anthocyanin synthesis were the primary metabolic pathways involved (Wang et al., 2023). The study by Du et al. (2024) further underscored the intricate relationship between the unique flavor of *Fragaria* species and key metabolites, including flavonoids, sugars, and amino acids (Du et al., 2024).

### 5.8 Comparisons and applications of analysis and recognition technologies

The techniques reviewed above operate at different levels of the strawberry aroma phenotype, providing complementary rather than interchangeable information. A detailed comparison of these techniques regarding analytical level, primary outputs, advantages, limitations, and suitable applications is summarized in Table 2. Sensory evaluation and E-nose characterize the overall aroma profile and perceptual quality; however, sensory evaluation is subjective and has low throughput, while E-nose is rapid and non-destructive but cannot identify individual compounds. HS-SPME-GC–MS, GC-IMS, and GC-O focus on the volatile chemical composition. HS-SPME-GC–MS offers strong qualitative and quantitative capabilities but is relatively time-consuming and not directly relevant to sensory analysis. GC-IMS enables rapid fingerprinting and discrimination but has weaker qualitative and quantitative capacities. GC-O directly links volatile compounds to olfactory perception and is essential for identifying key odorants, although it is subjective and labor-intensive. Genomics, transcriptomics, proteomics, and metabolomics provide mechanistic insights at the gene, transcript, protein, and metabolite levels, respectively. Genomics has high coverage and stability but cannot directly reflect dynamic aroma formation. Transcriptomics reveals dynamic gene expression but does not always correlate with protein abundance (Liu et al., 2023). Proteomics reflects functional molecules but faces challenges in coverage and quantification (Wang et al., 2025). Metabolomics is closest to the aroma phenotype but requires accurate metabolite annotation and robust data integration. Therefore, an integrated strategy combining sensory analysis, volatile profiling, and multi-omics is essential to link chemical detection, sensory perception, and molecular mechanisms, thereby supporting aroma-oriented breeding and postharvest quality management (Fan et al., 2022).

**Table 2:** Comparisons of analytical techniques for aroma detection.

Technique	Main output	Main advantages	Main limitations	Application scenarios	References
Sensory evaluation	Overall aroma perception	Direct reflection of human perception	Subjective, low throughput, panel training required	Sensory validation	(Oliver et al., 2018; Schwieterman et al., 2014; S. Xu et al., 2025)
E-nose	Global volatile fingerprint	Rapid, non-destructive, easy operation	Unable to identify individual compounds, poor quantification	Rapid classification, freshness assessment	(Rao et al., 2020; S. Xu et al., 2024)
HS-SPME-GC–MS	Identity and concentration of volatile compounds	High sensitivity, superior separation, qualitative and quantitative capacity	Time-consuming, matrix effect and fiber coating bias	VOC identification and quantification	(L. Xu et al., 2025; S. Xu et al., 2024; Zheng et al., 2023)
GC-IMS	Volatile fingerprint	Fast, high sensitivity, minimal sample pretreatment	Weak qualitative and quantitative performance, chemometrics-dependent	Rapid discrimination, marker screening	(S. Xu et al., 2024; S. Xu et al., 2025; Zhang et al., 2023; Zhang et al., 2025)
GC-O	Odor-active compounds	Direct linkage to olfactory perception	Subjective, labor-intensive, requires GC–MS coupling	Identification of key odorants	(Mahmoud & Zhang, 2024; Prat et al., 2014; Xiaofen et al., 2011)
Genomics	Genetic variations and genetic resources	Stable, high genomic coverage	Cannot reflect dynamic aroma formation	Gene mining, molecular breeding	(Barbey et al., 2021; Edger et al., 2019; Qiao et al., 2021)
Transcriptomics	Gene expression profiles	Dynamic monitoring, high throughput	mRNA abundance not always consistent with protein level	Candidate gene screening, regulatory network construction	(Baldwin et al., 2025; Barbey et al., 2021; Fang et al., 2024; Lu et al., 2021)
Proteomics	Protein abundance and post-translational modifications	Reflects functional effector molecules	Limited proteome coverage, quantification bottlenecks	Verification of enzymes and regulatory proteins	(Li et al., 2013; Lu et al., 2020; Song et al., 2015)
Metabolomics	Metabolite profiles	Close to phenotypic traits, comprehensive coverage	Difficult metabolite annotation, complex datasets	Pathway dissection, biomarker discovery	(Fang et al., 2024; Liu et al., 2024; Song et al., 2023; Wang et al., 2023)

Integrating multiple omics approaches offers a comprehensive understanding of strawberry aroma formation by linking genetic variation and gene regulation to protein abundance, metabolic changes, and volatile profiles. The value of this integration lies not merely in the combination of datasets, but in the connection of molecular changes across different biological levels to defined aroma-related phenotypes. Fan et al. (2022) established a multi-omics framework for cultivated strawberry by combining volatile phenotyping with genome-wide association analysis, expression QTL mapping, haplotype-resolved genomic data, and allele-specific expression analysis. This approach linked genetic variation to volatile traits and supported the involvement of anthranilate synthase alpha subunit 1 in methyl anthranilate biosynthesis through transient expression assays (Fan et al., 2022). At the developmental level, Lu et al. (2020) combined untargeted proteomic and metabolomic profiling across five fruit-development stages with gene expression analysis and functional experiments. Their findings supported a role for *FaMYB9* in C6 volatile biosynthesis and identified an interaction between *FaMYB9* and *FaLOX5*; silencing and overexpression of *FaMYB9* altered C6 volatile accumulation (Lu et al., 2020). Zheng et al. (2025) further combined transcriptomic and metabolomic analyses with *FaHMGR* overexpression and suppression experiments. Changes in terpenoid-related metabolites, including linalool, were associated with altered pathway activity, while *ERF118* and *WRKY12* were proposed as candidate regulatory factors (Zheng et al., 2025). Collectively, these studies illustrate how genetic, transcriptional, protein-level, and metabolite evidence can be interconnected to investigate aroma-related regulatory mechanisms. In addition to single-omics analysis, multi-omics integrative strategies are essential for elucidating the molecular mechanisms underlying the formation of strawberry aroma. Genomics provides insights into genetic variations and candidate genes, while transcriptomics reveals dynamic expression patterns. Proteomics confirms the abundance and activity of functional enzymes and regulatory factors, and metabolomics captures downstream metabolites and aroma precursors (Porter et al., 2023). By integrating these data with volatile profiling and GC-O, it is possible to link molecular changes to sensory-relevant aroma phenotypes. Integrative strategies, such as correlation network analysis, weighted gene co-expression network analysis, and multi-omics QTL mapping, can identify regulatory modules and biosynthetic pathways that control the accumulation of aroma compounds (Barbey et al., 2021; He et al., 2025; Wang et al., 2022). For instance, integrated transcriptomic and metabolomic analyses have elucidated genes and metabolites associated with ester and furanone biosynthesis in strawberries (Fang et al., 2024; Zheng et al., 2025).

Nevertheless, several limitations of multi-omics analysis in strawberry aroma research must be acknowledged. First, the coverage of proteomics remains insufficient, and most integrative studies predominantly rely on genomic, transcriptomic, and metabolomic data, thereby leaving post-transcriptional regulation and enzyme activity largely unexplored (Song et al., 2015). Second, the relationships among volatile metabolites, sensory perception, and consumer preference are often indirect, necessitating further sensory validation. Third, data integration is impeded by platform-specific biases, batch effects, missing values, limited sample sizes, and the absence of standardized analytical pipelines, which complicates cross-study comparisons (Mosley et al., 2024). Finally, causal inference from candidate genes to aroma phenotypes still requires functional validation, constrained by the complexity of the strawberry genome and cultivar-dependent transformation efficiency. Therefore, current multi-omics findings should be viewed as candidate regulatory networks rather than definitive causal mechanisms.

## 6 Conclusions and future perspectives

Strawberry aroma is a sensory phenotype determined by the composition, relative abundance, and interactions of volatile compounds, rather than merely reflecting the quantity of VOCs. Its variation is influenced by factors such as genotype, ripening stage, cultivation practices, postharvest handling, and the underlying biosynthetic and regulatory processes. The integration of OT, OAV, and GC-O with sensory validation allows for the differentiation between compounds that are merely ‘detected’ and those that are ‘truly perceived’ as key aroma-active components. In contrast, omics technologies, including transcriptomics and metabolomics, are primarily employed for mechanistic dissection and must be corroborated with chemical and sensory evidence. Future research should focus on the targeted quantification of volatile compounds and the functional validation of key genes and regulators such as *FaQR, FaNES1, FaFAD1* and MYB/ERF. Additionally, molecular analyses should be integrated with GC-O and sensory recombination or omission tests to identify molecular changes responsible for variations in aroma perception. This approach will provide a foundation for aroma-oriented breeding and postharvest quality management. Furthermore, the integration of multi-omics is essential for elucidating the synthesis and release mechanisms of aroma compounds and for constructing characteristic volatile fingerprints of strawberries. However, such multi-omics integration faces challenges, including complex data integration, sample heterogeneity, and the lack of standardized workflows. These challenges primarily arise from the need for different normalization strategies for heterogeneous multi-omics datasets, biological and environmental variation among samples, and inconsistent analytical and annotation protocols.

## CRediT authorship contribution statement

**Su Xu:** Writing – review & editing, Writing – original draft, Visualization, Funding acquisition, Conceptualization. **Jinjin Jiang:** Writing – original draft, Visualization. **Guangcan Tao:** Writing – review & editing. **Qiang Fei:** Writing – review & editing. **Lingshuai Meng:** Writing – review & editing. **Qinglong Meng:** Writing – review & editing. **Liang Zhao:** Writing – review & editing, Conceptualization.

## Declaration of competing interest

The authors declare that they have no known competing financial interests or personal relationships that could have appeared to influence the work reported in this paper.

## Data Availability

No new data were created or analyzed in this study. Data sharing does not apply to this article.
